# Psychological, Political, and Situational Factors Combine to Boost COVID-19 Conspiracy Theory Beliefs

**DOI:** 10.1017/S000842392000058X

**Published:** 2020-06-11

**Authors:** Joanne M. Miller

**Affiliations:** Department of Political Science and International Relations, University of Delaware, 347 Smith Hall, 18 Amstel Ave, Newark, DE 19716, USA

## Abstract

Conspiracy theories (CTs) are not solely the domain of extremists and paranoids. They cut across demographic and political differences (Uscinski and Parent, 2014) and can have negative social/political consequences. For example, Imhoff and Lamberty (2020) find that belief that the seriousness of COVID-19 is being exaggerated is negatively correlated with self-reported preventative behaviours such as hand washing and social distancing, and belief that the virus was intentionally created by humans is positively correlated with self-reported hoarding of food, sanitary products, and gasoline/oil, as well as stocking up on weapons.

Conspiracy theories (CTs) are not solely the domain of extremists and paranoids. They cut across demographic and political differences (Uscinski and Parent, 2014) and can have negative social/political consequences. For example, Imhoff and Lamberty (2020) find that belief that the seriousness of COVID-19 is being exaggerated is negatively correlated with self-reported preventative behaviours such as hand washing and social distancing, and belief that the virus was intentionally created by humans is positively correlated with self-reported hoarding of food, sanitary products, and gasoline/oil, as well as stocking up on weapons.

Scholars have identified psychological, political, and situational factors that contribute to the likelihood that someone will endorse a CT. On the psychological side, people who have a tendency to explain events as the result of a conspiracy (conspiratorial thinking) are more likely to endorse specific CTs than their counterparts (Uscinski and Parent, 2014). On the political side, CTs can help people confirm/bolster their political worldviews: Democrats/liberals are more likely to endorse CTs that make Republicans/conservatives look bad, and vice versa (Miller et al., 2016). Finally, situations that give rise to uncertainty can lead to CT beliefs (van Prooijen and Acker, 2015).

The global COVID-19 pandemic is a perfect storm for activating these psychological, political, and situational factors. Uscinski et al. (2020) find that conspiratorial thinking is positively related to the endorsement of two COVID-19 CTs. Moreover, because the current political context is one in which a Republican president is being widely criticized for his handling of the pandemic (threatening the political worldviews of those on the right), Republicans are more likely to believe COVID-19 CTs than Democrats or Independents (Uscinski et al., 2020). Finally, personal uncertainty (likely induced by the pandemic) is positively related to COVID-19 CT beliefs (Miller, 2020).

To date, research on the antecedents of CT beliefs has treated each factor as having an independent effect. Some scholars argue that psychological and political factors dominate, regardless of the situation. Others argue that some situations give rise to CT beliefs across the board, regardless of individual-level psychological predispositions or political attitudes/identities. In contrast, I argue that individual and situational factors *interact* to amplify CT beliefs to a greater extent than any single factor does on its own. People who are dispositionally-inclined to view the world in conspiratorial terms do not explain *every* event (not even every big negative event) as being the result of a conspiracy. Partisans/ideologues do not *always* endorse CTs that make the other side look bad, and *not everyone* who feels uncertain in response to a negative event endorses CTs to try to alleviate the uncertainty. The current research demonstrates – for the first time – how these psychological, political, and situational factors interact with one another to amplify CT beliefs in the context of the COVID-19 pandemic.

## Methods

U.S. adults (n = 3,019) completed an online survey between April 24 and April 28 via Lucid Theorem. Lucid provides a quota sample (using U.S. Census benchmarks) of individuals who have agreed to participate in scientific research. Data were also weighted based on the 2018 Current Population Survey (CPS) benchmarks for education, income, sex, race, and ethnicity to more accurately represent the U.S. population (see Appendix A, available online, for a comparison of sample characteristics with the CPS).

The survey measured belief in the 11 COVID-19 CTs; the order of the CTs was randomized across respondents. Responses were assessed using a standard four-point format, coded from 1–4 with higher numbers representing greater belief (see Appendix B, available online, for question wording/coding for all variables included in the analyses). A COVID-19 CT Belief Index was created by averaging the 11 CTs (Cronbach's Alpha = .86).

Table 1 reports the question wordings and percent of respondents who believe each CT.

**Table 1:** COVID-19 CTs

Variable label	Question wording	Percent who believe the CT is definitely or probably true
AccChina	The virus was accidentally released by China.	52
BioChina	The virus is a biological weapon intentionally released by China.	49
Gates	Former Microsoft CEO Bill Gates is creating a tracking device to be injected with the coronavirus vaccine.	46
Media	The media are exaggerating the seriousness to make President Trump look bad.	43
Tests	Democratic governors are not distributing coronavirus tests to make President Trump look bad.	41
WorldPop	The coronavirus was intentionally created to reduce the world's population.	38
Hoard	Democratic governors are hoarding ventilators to make President Trump look bad.	36
Scientists	Scientists are exaggerating the seriousness to make President Trump look bad.	31
5GTech	5G technology is causing the coronavirus to spread faster.	22
AccUS	The virus was accidentally released by the US.	19
NotReal	The coronavirus isn't real.	8

Partisanship was measured with two dummy variables. *Republican* was coded such that Republicans/Republican leaners = 1 and everyone else = 0. *Independent* was coded such that Independents = 1 and everyone else = 0.

*Conspiratorial thinking* was measured via four items (averaged; Uscinski et al., 2020) that asked respondents how much they agreed or disagreed (on five-point scales) with statements such as “Much of our lives are being controlled by plots hatched in secret places.”

*Personal uncertainty* was measured via three items (averaged) that assessed how uncertain people currently felt about: (1) themselves, (2) their place in the world, and (3) their future, at this very moment (five-point scales ranging from “not at all uncertain” to “extremely uncertain”).

*Resilience* was measured via six items (averaged; Smith et al., 2008) that asked respondents how much they agreed or disagreed (on five-point scales coded such that higher numbers = greater resilience) with statements such as: “I tend to bounce back quickly after hard times.”

Age, sex, education, income, race, and ethnicity were included as controls. All independent variables were re-coded to range from 0–1.

## Results

### COVID-19 CT Beliefs Are Prevalent

As Table 1 shows, a striking percentage endorse COVID-19 CTs (see also Uscinski et al., 2020). At the high end, 52 per cent believe the virus was accidentally released by China, 49 per cent believe it is a Chinese biological weapon, and 46 per cent believe that Bill Gates is probably or definitely creating a tracking device to be injected along with the COVID vaccine. At the low end, very few people (8%) believe that the virus is not real.

### Partisanship, Conspiratorial Thinking, and Personal Uncertainty Are Positively Related and Resilience Is Negatively Related to COVID-19 CT Beliefs

**Figure 1:**
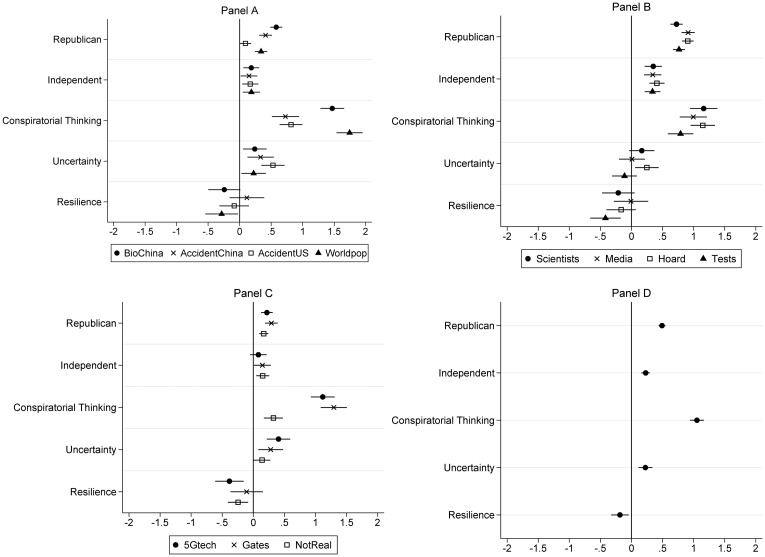
Predictors of COVID-19 conspiracy theories.

*Note:* This figure reports the unstandardized regression coefficients (all variables are coded to range from 0 to 1) with 95 per cent confidence intervals from ordinary least squares (OLS) models that include controls for age, education, income, gender, race, and ethnicity. The dependent variables are beliefs in the individual COVID-19 CTs (Panels A–C) and scores on a COVID-19 CT Belief Index composed of the average of the 11 CTs (Panel D). The larger the absolute value of the coefficient (as in, the farther a dot is to the left or right of the vertical line, 0), the stronger the relationship between the independent variable (on the left) and each COVID-19 CT belief. Dots to the right of the line indicate a positive relationship; dots to the left of the line indicate a negative relationship. See Tables A1 and A2 in Appendix C, available online, for the full models.

Across the 11 individual CTs, the psychological predisposition to view the world in conspiratorial terms (conspiratorial thinking) is a consistent, positive predictor of the individual CTs (see Panels A–C of Figure 1). The greater the tendency for people to view events as the product of a conspiracy, the more likely they are to endorse the CTs. Political and situational variables also matter. Compared to Democrats, Republicans and Independents are more likely to believe COVID-19 CTs. Personal uncertainty is also positively related to most of the CTs. Another dispositional variable—resilience—seems to serve as a bit of a buffer. People higher in resilience are less likely to believe over half of the CTs.

Conspiratorial thinking and uncertainty are positive predictors of the COVID-19 CT Belief Index, whereas resilience is a negative predictor (see Panel D of Figure 1). Republicans and Independents both score higher on the belief index than Democrats. Of these variables, conspiratorial thinking is the strongest predictor.

### The Positive Effect of Conspiratorial Thinking Is Stronger for People Who Currently Feel Uncertain about Their Lives

To provide more nuance to our understanding of why people believe COVID-19 CTs, I test whether a relatively stable characteristic of individuals—the degree to which people tend to explain events as the result of a conspiracy—interacts with something that can vary depending on the current situation—uncertainty about oneself, one's future, and one's place in the world. In other words, we know that people higher in conspiratorial thinking and people who are higher in uncertainty are more likely to believe COVID-19 CTs (see Figure 1). The question I ask here is: Does pandemic-induced uncertainty make it *more* likely that people who are already predisposed to view the world in conspiratorial terms will endorse COVID-19 CTs than their counterparts for whom the pandemic has not increased personal uncertainty?

**Figure 2:**
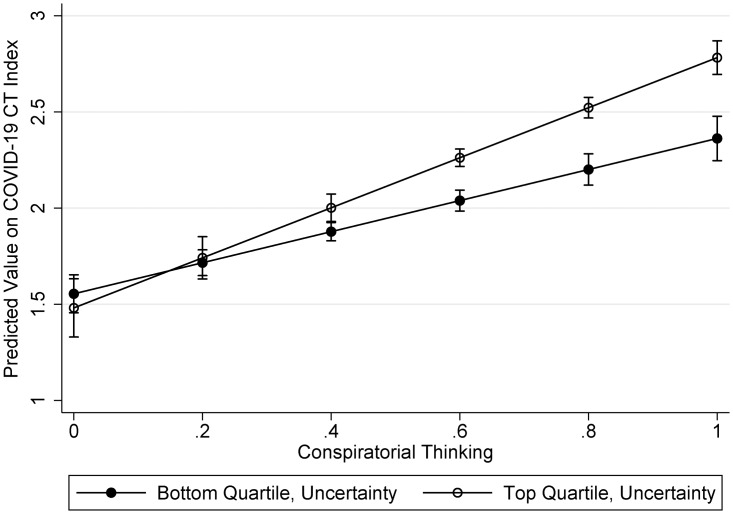
Interaction between conspiratorial thinking and uncertainty.

*Note:* This figure displays the effect of conspiratorial thinking for people who report the lowest and highest levels of personal uncertainty. The steeper the line, the bigger the effect of a one-unit increase in conspiratorial thinking on the COVID-19 CT Index. See Model 1, Table A3 in Appendix C, available online, for the full model.

To assess whether the effect of conspiratorial thinking is stronger for people higher in personal uncertainty, a variable to represent the interaction between conspiratorial thinking and uncertainty was added to the model reported in Panel D of Figure 1. This interaction was statistically significant (b = .92, p < .001; see Model 1, Table A3 in Appendix C, available online). To demonstrate the shape of the interaction, Figure 2 reports the effect of conspiratorial thinking on the COVID-19 CT belief index for people who scored in the bottom and top quartiles of personal uncertainty. As expected, among people who feel the *least* uncertain, conspiratorial thinking has a positive effect on the COVID-19 CT index (i.e., the greater the predisposition toward CTs in general, the greater the belief in COVID-19 CTs). However, among people who feel the *most* uncertain, conspiratorial thinking has an even stronger positive effect (as represented by the steeper line). In sum, a situation like a pandemic that arouses feelings of uncertainty amplifies the effect of conspiratorial thinking on COVID-19 CT beliefs.

### The Interaction Between Conspiratorial Thinking and Uncertainty Obtains for Republicans but Not for Democrats

**Figure 3:**
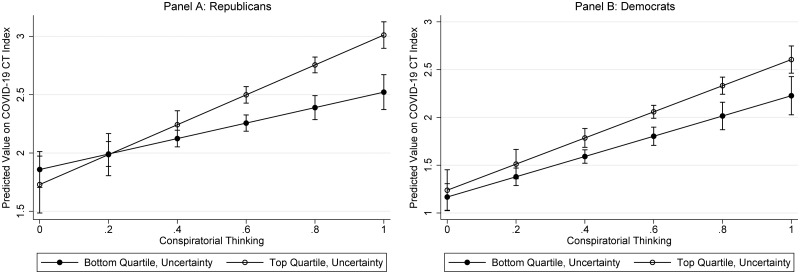
Interaction between conspiratorial thinking and uncertainty for Republicans and Democrats.

*Note:* Panel A displays the effect of conspiratorial thinking for Republicans who report the lowest and highest levels of personal uncertainty. Panel B displays the effect of conspiratorial thinking for Democrats who report the lowest and highest levels of personal uncertainty. The steeper the line, the bigger the effect of a one-unit increase in conspiratorial thinking on the COVID-19 CT Index. See Model 2, Table A3 in Appendix C, available online, for the full model.

Given that Republicans are more likely than Democrats to believe COVID-19 CTs to protect their political worldviews (see Figure 1), it might also be the case that uncertainty activates conspiratorial thinking to a greater extent for Republicans than Democrats. To test this hypothesis, I added a three-way interaction between conspiratorial thinking, uncertainty, and a partisanship dummy variable (Republican = 1; Democrat = 0) to Model 1, Table A3 in Appendix C (available online). The three-way interaction was marginally statistically significant (b = .65, p < .10; see Model 2, Table A3 in Appendix C). To demonstrate the shape of the interaction, Figure 3 reports the effect of conspiratorial thinking for people who scored in the bottom and top quartiles of personal uncertainty separately for Republicans and Democrats. For Republicans, the pattern depicted in Figure 3 holds; among Republicans who feel the most uncertain (compared to their less uncertain counterparts), conspiratorial thinking has a stronger effect on COVID-19 beliefs. The same is not the case for Democrats. The effects of conspiratorial thinking among more and less uncertain Democrats are not different from one another (as demonstrated by the nearly perfectly parallel lines in Panel B).

## Discussion

In sum, uncertainty induced by COVID-19 strengthens the effect of conspiratorial thinking on belief in a wide array of COVID-19 CTs. This multiplicative effect of uncertainty and conspiratorial thinking occurs only for Republicans—people whose political worldview is being threatened by criticisms of a Republican president's handling of the pandemic. The current research also finds that a heretofore unexplored psychological predisposition—resilience—is a buffer against COVID-19 CTs.

Together, these findings suggest that the best path forward to mitigating CT beliefs may be to provide people with information and tools to enable them to be resilient in the face of uncertainty (Nyhan and Reifler, 2019; van Prooijen, 2018), and to amplify corrective information by co-partisans to dilute the power of partisan-motivated reasoning (Benegal and Scruggs, 2018; Berinsky, 2015; Uscinski et al., 2020). Because the relatively stable predisposition toward conspiratorial thinking in and of itself is a powerful predictor of COVID-19 CTs, attempts at mitigating these beliefs face an uphill battle. The interactive effect of conspiratorial thinking, partisanship, and situationally induced uncertainty presents an even more formidable foe. Corrective attempts that confront logical fallacies or provide factual information (Vraga et al., 2020) may be effective in the short term. But if the feelings of uncertainty that activate psychological and political predispositions remain intact, so does the motivation to alleviate those feelings. Another CT always seems to be waiting in the wings as a tempting antidote.

## Supplementary material

For supplementary material accompanying this paper visit http://dx.doi.org/10.1017/S000842392000058X. click here to view supplementary material

## References

[ref1] Benegal, Salil D., and Lyle A. Scruggs. 2018 “Correcting Misinformation about Climate Change: The Impact of Partisanship in an Experimental Setting.” Climatic Change 148 (1): 61–80.

[ref2] Berinsky, Adam. 2015 “Rumors and Health Care Reform: Experiments in Political Misinformation.” British Journal of Political Science 47 (2): 241–62.

[ref3] Imhoff, Roland and Pia Lamberty. 2020 “A Bioweapon or a Hoax? The Link Between Distinct Conspiracy Beliefs about the Coronavirus Disease (COVID-19) Outbreak and Pandemic Behavior.” Social Psychological and Personality Science, in press. https://psyarxiv.com/ye3ma/10.1177/1948550620934692 PMC7342934 38602949

[ref4] Miller, Joanne M. 2020 “Do COVID-19 Conspiracy Theories Form a Monological Belief System?” Canadian Journal of Political Science. Advance online publication. doi: 10.1017/S0008423920000517

[ref5] Miller, Joanne M., Kyle L. Saunders, and Christina E. Farhart. 2016 “Conspiracy Endorsement as Motivated Reasoning: The Moderating Roles of Political Knowledge and Trust.” American Journal of Political Science 60 (4): 824–44.

[ref6] Nyhan, Brendan, and Jason Reifler. 2019 “The Roles of Information Deficits and Identity Threat in the Prevalence of Misperceptions.” *Journal of Elections*, Public Opinion and Parties 29 (2): 222–44.

[ref7] Smith, Bruce W., Jeanne Dalen, Kathryn Wiggins, Erin Tooley, Paulette Christopher, and Jennifer Bernard. 2008 “The Brief Resilience Scale: Assessing the Ability to Bounce Back.” International Journal of Behavioral Medicine 15 (3): 194–200. 18696313 10.1080/10705500802222972

[ref8] Uscinski, Joseph E., Adam M. Enders, Casey M. Klofstad, Michelle Seelig, John Funchion, Caleb Everett, Stephan Wuchty, Kamal Premaratne and Manohar Murthi. 2020 “Why Do People Believe COVID-19 Conspiracy Theories?” The Harvard Kennedy School (HKS) Misinformation Review. 10.37016/mr-2020-015.

[ref9] Uscinski, Joseph E., and Joseph M. Parent. 2014 American Conspiracy Theories. Oxford: Oxford University Press.

[ref10] van Prooijen, Jan-Willem. 2018 “Empowerment as a Tool to Reduce Belief in Conspiracy Theories” In Conspiracy Theories and the People Who Believe Them, ed. Joseph E. Uscinski. New York: Oxford University Press.

[ref11] van Prooijen, Jan-Willem, and Michelle Acker. 2015 “The Influence of Control on Belief in Conspiracy Theories: Conceptual and Applied Extensions.” Applied Cognitive Psychology 29 (5): 753–61.

[ref12] Vraga, Emily K., Sojung Claire Kim, John Cook, and Leticia Bode. 2020 “Testing the Effectiveness of Correction Placement and Type on Instagram.” International Journal of Press and Politics. Advance online publication.: doi:10.1177/1940161220919082

