# Supplementary material for: Psychological, Political, and Situational Factors Combine to Boost COVID-19 Conspiracy Theory Beliefs
Source: Can J Polit Sci. 2020 Jun 11:1–8. doi: 10.1017/S000842392000058X (PMC7338396; doi:10.1017/S000842392000058X)
Supplement: Supplementary file 1 [file S000842392000058Xsup001.docx]

**Supplementary Materials for**

**Psychological, Political, and Situational Factors Combine to Boost COVID-19 Conspiracy Theory Beliefs**

Joanne M. Miller

Associate Professor, Political Science and International Relations

University of Delaware

June 8, 2020

**Appendix A. Comparison of Raw and Weighted Lucid Data to Current Population Survey (U.S. Census 2018) Benchmarks**

|  |  |  |  |
| --- | --- | --- | --- |
|  | Raw Data | Weighted Data | CPS 2018 |
| Female | 53% | 51% | 51% |
| College Degree | 44% | 35% | 31% |
| Black/Non-Hispanic | 11% | 12% | 13% |
| White/Non-Hispanic | 69% | 61% | 62% |
| Hispanic | 11% | 15% | 18% |
| Age (mean) | 46 | 45 | 37 |
| Income (median) | $40-44,999 | $55-59,999 | $55-59,999 |

Note: Weights adjust for gender, education, race, ethnicity, and income.

**Appendix B. Question Wordings and Variable Coding**

**Conspiracy Theories**

All conspiracy theory questions were coded to range from 1-4, with higher numbers representing greater belief.

[BioChina] Some people believe that the coronavirus is actually a biological weapon that was released from a laboratory in China. Others do not believe this. What do you think?
The coronavirus is: definitely a Chinese biological weapon; probably a Chinese biological weapon; probably not a Chinese biological weapon; definitely not a Chinese biological weapon.

[AccChina] Some people believe that the coronavirus was accidentally released from a laboratory in China. Others do not believe this. What do you think? The coronavirus was: definitely accidentally released from a Chinese lab; probably accidentally released from a Chinese lab; probably not accidentally released from a Chinese lab; definitely not accidentally released from a Chinese lab.

[AccUS] Some people believe that the coronavirus was accidentally released from a laboratory in the United States. Others do not believe this. What do you think? The coronavirus was: definitely accidentally released from a U.S. lab; probably accidentally released from a U.S. lab;

probably not accidentally released from a U.S. lab; definitely not accidentally released from a U.S. lab.

[Scientists] Some people believe that fear-mongering scientists are intentionally exaggerating the seriousness of the coronavirus to make President Trump look bad. Others do not believe this. What do you think? Scientists are: definitely exaggerating the seriousness of the coronavirus; probably exaggerating the seriousness of the coronavirus; probably not exaggerating the seriousness of the coronavirus; definitely not exaggerating the seriousness of the coronavirus.

[Media] Some people believe that fear-mongering media outlets are intentionally exaggerating the seriousness of the coronavirus to make President Trump look bad. Others do not believe this. What do you think? Media outlets are: definitely exaggerating the seriousness of the coronavirus; probably exaggerating the seriousness of the coronavirus; probably not exaggerating the seriousness of the coronavirus; definitely not exaggerating the seriousness of the coronavirus.

[NotReal] Some people believe that the coronavirus isn't real, and that doctors and scientists are in on the elaborate hoax. Others do not believe this. What do you think? The coronavirus: definitely is not real; probably is not real; probably is real; definitely is real.

[Hoard] Some people believe that Democratic Governors are hoarding ventilators to make President Trump look bad. Others do not believe this. What do you think? Democratic Governors are: definitely hoarding ventilators; probably hoarding ventilators; probably not hoarding ventilators; definitely not hoarding ventilators.

[Tests] Some people believe that Democratic Governors are intentionally not distributing the coronavirus tests the federal government has given them to make President Trump look bad. Others do not believe this. What do you think? Democratic Governors are: definitely not distributing all the coronavirus tests they have; probably not distributing all the coronavirus tests they have; probably distributing all the coronavirus tests they have; definitely distributing all the coronavirus tests they have.

[Tech] Some people believe that 5G technology is causing the coronavirus to spread faster. Others do not believe this. What do you think? 5G technology is: definitely causing the coronavirus to spread faster; probably causing the coronavirus to spread faster; probably not causing the coronavirus to spread faster; definitely not causing the coronavirus to spread faster.

[Gates] Some people believe former Microsoft CEO Bill Gates is creating a tracking device to be injected with the coronavirus vaccine. Others do not believe this. What do you think? Bill Gates is: definitely creating a tracking device for the vaccine; probably creating a tracking device for the vaccine; probably not creating a tracking device for the vaccine; definitely not creating a tracking device for the vaccine.

[WorldPop] Some people believe that the coronavirus was intentionally created as a plot to reduce the world's population. Others do not believe this. What do you think? The coronavirus was: definitely created to reduce the world's population; probably created to reduce the world's population; probably not created to reduce the world's population; definitely not created to reduce the world's population.

[COVID-19 CT Index] An index was created by averaging responses to the 11 CTs described above. Responses could range from 1-4, with higher numbers representing greater belief in COVID-19 CTs.

**Party Identification**

Generally speaking, do you usually think of yourself as a *Democrat, a Republican, an Independent, or what*? Democrats and Republicans branched to: Would you call yourself a *strong Democrat [Republican]* or a *not very strong Democrat [Republican]*? Independents and others branched to: Do you think of yourself as *closer to the Democratic Party* or *closer to the Republican Party*?

A Republican dummy variable was created such that 1 = Republican or lean Republican and 0 = Democrat/lean Democrat and pure Independents. An Independent dummy variable was created such that 1 = pure Independents and 0 = Republicans/leaners and Democrats/leaners.

**Conspiratorial Thinking**

Responses to the 4 items were coded to range from 0-1 (with higher numbers meaning more conspiratorial) and then averaged (alpha = .81).

How much do you agree or disagree with the following statements? Strongly agree, somewhat agree, neither agree nor disagree, somewhat disagree, strongly disagree.

Much of our lives are being controlled by plots hatched in secret places.

Even though we live in a democracy, a few people will always run things anyway.

The people who really 'run' the country are not known to the voters.

Big events like wars, economic recessions, and the outcomes of elections are controlled by small groups of people who are working in secret against the rest of us.

**Personal Uncertainty**

Responses to the 3 items were coded to range from 0-1 (with higher numbers meaning more uncertainty) and then averaged (alpha=.87). Response options were: extremely uncertain, very uncertain, somewhat uncertain, not too uncertain, not uncertain at all.

At this very moment, how uncertain do you feel about yourself?

At this very moment, how uncertain do you feel about your place in the world?

At this very moment, how uncertain do you feel about your future?

**Resilience**

Responses to the 6 items were coded to range from 0-1 (with higher numbers meaning more resilience) and then averaged (alpha=.75).

Now we have a brief set of questions concerning how people feel. After reading each item, please respond as to how closely you agree or disagree with how each item describes you and your feelings about yourself. Strongly disagree, somewhat disagree, neither agree nor disagree, somewhat agree, strongly agree.

I tend to bounce back quickly after hard times.

I have a hard time making it through stressful events (reverse-coded).

It does not take me long to recover from a stressful event.

It is hard for me to snap back when something bad happens (reverse-coded).

I usually come through difficult times with little trouble.

I tend to take a long time to get over set-backs in my life (reverse-coded).

**Age**

Respondent age ranged from 18-88 and was recoded to range from 0-1.

**Female**

A dummy variable [Female] was created such that 0=male and 1=female.

**Education**

Education was coded into 4 categories ranging from 0-1: less than high school, high school graduate, some college, and college graduate or more.

**Income**

Income was coded into quartiles ranging from 0-1: less than or equal to $19,999, $20,000-44,999, $45,000-79,999, and $80,000 or more

**White**

A dummy variable [White] was created such that 0=not White and 1=White.

**Hispanic**

A dummy variable [Hispanic] was created such that 0=not Hispanic and 1= Hispanic.

**Appendix C. Full Tables**

Table A1. Main Effect Predictors of Individual COVID-19 Conspiracy Theories

Note: Table entries are unstandardized regression coefficients. Standard errors appear in parentheses.

+ p<.10 *p<.05 **p<.01 ***p<.001

Table A2. Predictors of COVID-19 Conspiracy Theory Index

|  | COVID-19 CT Index |
| --- | --- |
| Republican | .49*** |
|  | (.03) |
|  |  |
| Independent | .23*** |
|  | (.04) |
|  |  |
| Consp. Thinking | 1.05*** |
|  | (.06) |
|  |  |
| Uncertainty | .22*** |
|  | (.06) |
|  |  |
| Resilience | -.19* |
|  | (.07) |
|  |  |
| Age | -.26*** |
|  | (.05) |
|  |  |
| Female | -.11*** |
|  | (.02) |
|  |  |
| Education | -.05 |
|  | (.04) |
|  |  |
| Income | -.05 |
|  | (.04) |
|  |  |
| White | -.13*** |
|  | (.03) |
|  |  |
| Hispanic | .09* |
|  | (.04) |
|  |  |
| Constant | 1.58*** |
|  | (.08) |
| *N* | 2760 |
| *R*^2^ | .39 |

Note: Table entries are unstandardized regression coefficients. Standard errors appear in parentheses.

+ p<.10 *p<.05 **p<.01 ***p<.001

Table A3. Conspiratorial Thinking X Uncertainty and Conspiratorial Thinking X Uncertainty X Partisanship (Republicans vs. Democrats) Predicting the COVID-19 CT Index

|  | Model 1 | Model 2 |
| --- | --- | --- |
| Conspiratorial Thinking | .68*** | .94*** |
|  | (.09) | (.14) |
|  |  |  |
| Uncertainty | -.37** | -.15 |
|  | (.12) | (.17) |
|  |  |  |
| Consp. Thinking X Uncertainty | .92*** | .58+ |
|  | (.18) | (.30) |
|  |  |  |
| Republican | .49*** |  |
|  | (.03) |  |
|  |  |  |
| Independent | .22*** |  |
|  | (.03) |  |
|  |  |  |
| Republican v. Democrat |  | .75*** |
|  |  | (.10) |
|  |  |  |
| RepvDem X Consp. Thinking |  | -.45* |
|  |  | (.19) |
|  |  |  |
| RepvDem X Uncertainty |  | -.43+ |
|  |  | (.25) |
|  |  |  |
| Consp. Thinking X Uncert X RepvDem |  | .65+ |
|  |  | (.39) |
|  |  |  |
| Resilience | -.21** | -.19* |
|  | (.07) | (.08) |
|  |  |  |
| Age | -.27*** | -.28*** |
|  | (.05) | (.05) |
|  |  |  |
| Female | -.10*** | -.11*** |
|  | (.02) | (.03) |
|  |  |  |
| Education | -.07+ | -.08 |
|  | (.04) | (.05) |
|  |  |  |
| Income | -.04 | -.03 |
|  | (.04) | (.04) |
|  |  |  |
| White | -.13*** | -.11** |
|  | (.03) | (.04) |
|  |  |  |
| Hispanic | .09* | .08+ |
|  | (.04) | (.05) |
|  |  |  |
| Constant | 1.84*** | 1.65*** |
|  | (.09) | (.11) |
| *N* | 2760 | 2353 |
| *R*^2^ | .40 | .42 |

Note: Table entries are unstandardized regression coefficients. Standard errors appear in parentheses.

+ p<.10 *p<.05 **p<.01 ***p<.001
